# Science in the Learning Gardens (SciLG): a study of students’ motivation, achievement, and science identity in low-income middle schools

**DOI:** 10.1186/s40594-018-0104-9

**Published:** 2018-03-26

**Authors:** Dilafruz R. Williams, Heather Brule, Sybil S. Kelley, Ellen A. Skinner

**Affiliations:** 1 Leadership for Sustainability Education, Graduate School of Education, Portland State University, Portland, OR 97201 USA; 2 Psychology, College of Liberal Arts & Sciences, Portland State University, Portland, OR 97201 USA

**Keywords:** Science education, Motivation, Academic outcomes, Middle school, School gardens, Science identity

## Abstract

**Background:**

Science in the Learning Gardens (henceforth, SciLG) program was designed to address two well-documented, inter-related educational problems: under-representation in science of students from racial and ethnic minority groups and inadequacies of curriculum and pedagogy to address their cultural and motivational needs. Funded by the National Science Foundation, SciLG is a partnership between Portland Public Schools and Portland State University. The sixth- through eighth-grade SciLG curriculum aligns with Next Generation Science Standards and uses school gardens as the milieu for learning. This provides the context to investigate factors that support success of a diverse student population using the motivational framework of self-determination theory.

**Results:**

This study reports results from 113 students and three science teachers from two low-income urban middle schools participating in SciLG. Longitudinal data collected in spring of sixth grade in 2015 and fall of seventh grade in 2015 for the same set of students included a measure of students’ overall motivational experiences in the garden (that combined their reports of relatedness, competence, autonomy, and engagement and teacher-reports of re-engagement in garden-based learning activities) to predict four science outcomes: engagement, learning, science grades, and science identity. Findings suggest that garden-based activities show promise for supporting students’ engagement and learning in science classes and in fostering students’ interest in pursuing science long-term.

**Conclusions:**

As concern for social justice is growing based on the underachievement of students from minority groups, resurgence of the school garden movement over the last several decades provides an opportunity to tip the scales by engaging students in authentic, real-world learning of science and cultivating their interests in science with holistic garden-based learning. This study highlights the role of students’ views of themselves as competent, related, and autonomous in the garden, as well as their engagement and re-engagement in the garden, as potential pathways by which garden-based science activities can shape science motivation, learning, and academic identity in science. Findings also suggest that the motivational model based on self-determination theory can be useful in identifying some of the “active ingredients”—in pedagogy, curriculum, and social relationships—that engage students in these garden-integrated science learning activities.

**Electronic supplementary material:**

The online version of this article (10.1186/s40594-018-0104-9) contains supplementary material, which is available to authorized users.

There is growing concern among policy-makers and practitioners in the USA that despite demographic trends showing increasing population growth among ethnic and racial minority groups (henceforth, minority), some of these groups—specifically, those who identify as African-American, Black, Hispanic, Latino, and Native-American—continue to be underrepresented in Science, Technology, Engineering, and Mathematics (STEM) majors in colleges and in STEM careers and professions (Elliott 2015; Museus et al. 2011; National Research Council [NRC] 2011; Quinn and Cooc 2015). A robust body of research highlights inadequacies in the teaching received by students in minority groups, resulting in a widening achievement gap between non-White and White students at all grade levels in schools (Bingham and Okagaki 2012; Brown and Crippen 2017; Howard 2012). Systemic gaps in access to high-quality STEM teaching and programming disproportionately impact students in low-income and racial minority groups (Chittum et al. 2017; Elliott 2015; Milner 2012; Stiles 2016). These disparities are especially troubling since research shows that marginalization and disengagement from STEM learning starts early, and if students lose interest and do not develop connections to these subjects by the end of middle school, they are less likely to pursue them in higher education (Elliott 2015; Fraser et al. 2011).

To address these concerns, scholars have called for culturally responsive pedagogy (Babco 2003; Gay 2000; Howard 2012), real-life active learning (Hawkins 2014; Howard 2012; Hrabowski and Maton 2009; Williams and Brown 2012; Yager and Brunkhorst 2014), and social contexts that facilitate motivation, engagement, and the development of a positive academic identity (Bircan and Sungur 2016; Skinner and Pitzer 2012; Skinner et al. 2008). Thus, critical to advancing STEM education for students in minority groups is to engage students with real-life issues via academically challenging learning activities within motivationally supportive social contexts. Garden-based educational programs—often known as Learning Gardens—use school gardens as the milieu for academic learning (Williams and Dixon 2013) and provide an important venue for engaging students in minority groups in science learning activities, in simple yet meaningful ways.

A program funded by the National Science Foundation, called Science in the Learning Gardens (henceforth, SciLG), was designed to address the needs of youth as well as to investigate how school gardens might offer a supportive milieu in which to engage them for success and positive outcomes in science. The research reported here draws upon this program, which provides a garden-based curriculum and instruction for sixth- through eighth-grade students, offered in partnership between Portland State University and Portland Public Schools. The SciLG team, comprised of university faculty and researchers, graduate students, and middle school science teachers, utilized a design-based approach to develop a garden-based science curriculum aligned with the Next Generation Science Standards (NGSS Lead States 2013). Design-based research takes a pragmatic approach to research and curriculum design through an iterative process of development, implementation, testing, and refinement. Formative information provides continual feedback for ongoing improvement (Barab and Squire 2004; Design-Based Research Collective 2003). This design-based approach reached into all aspects of the SciLG curriculum design, including instructional planning and assessment, with the team of faculty and teachers working closely to ensure alignment of SciLG to explicitly address NGSS performance expectations while keeping adolescent development at the forefront of the project.

Informed by principles of culturally responsive pedagogy, SciLG uses school gardens as contexts for hands-on, experiential, and holistic science learning activities. The program also draws upon the motivational framework of self-determination theory (SDT; Ryan and Deci 2016, 2017) in offering curricular and instructional experiences that meet students’ fundamental psychological needs for relatedness, competence, and autonomy in their academic work. In so doing, SciLG intends to support the development of students’ science identity, science engagement, science learning, and achievement. For this study, we focused on two research questions: (R1) *Concurrent effects of garden experiences on science outcomes*: Are students’ motivational experiences (of relatedness, competence, autonomy, and engagement and re-engagement) in SciLG gardening activities connected to four science outcomes (science identity, science-class engagement, science learning, and science grades)? and (R2) *Longitudinal effects of garden experiences on science outcomes*: Do students’ motivational experiences in SciLG gardening activities in the spring term of sixth grade predict the four science outcomes in the subsequent academic year—namely, fall term of seventh grade?

## Science in the Learning Gardens (SciLG) program

### Curriculum and instruction

The SciLG curriculum (http://learning-gardens.org/) addresses the three dimensions of science education called for in the *Framework for K-12 Science Education* (National Research Council [NRC] 2012) and the NGSS (NGSS Lead States 2013)*—*disciplinary core ideas, cross-cutting concepts, and the practices of science and engineering. SciLG connects key concepts in the NGSS with a middle school science curriculum, while simultaneously integrating school gardens as a context for meaningful and high-quality science learning activities, providing opportunities for students to engage in practices of science and engineering. Instructional units incorporate issues such as the impacts of climate change on local food systems. Contextualizing large, complex issues in a local setting allows students to engage in scientific endeavors in meaningful ways. Because of the increased emphasis on engineering design in the *Framework for K-12 Science Education* (National Research Council [NRC]. 2012) and the NGSS (NGSS Lead States 2013), and because challenges routinely emerge in gardens, a key emphasis throughout the SciLG curriculum has been *problem-solving* (see Additional file 1 for examples).

**Fig. 1:**
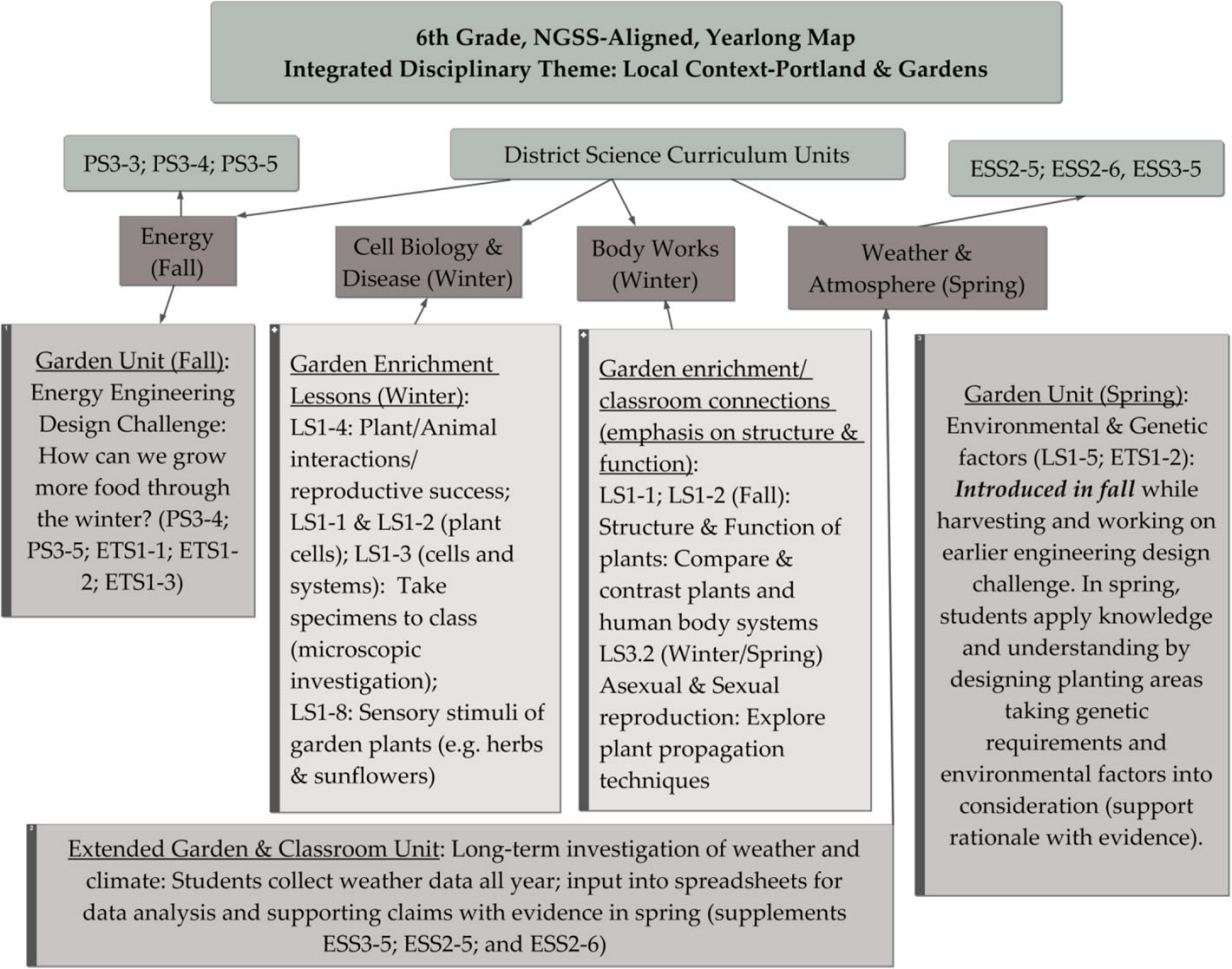
Yearlong curriculum map, co-created with collaborating teachers outlining learning garden activities and alignment to classroom curriculum. Three units align with the district-adopted science curriculum, and additional enrichment lessons provide hands-on context and application for disciplinary concepts. By emphasizing extended units of instruction, this curriculum can be used for application and enrichment of school curriculum, and/or as stand-alone garden curriculum. (NGSS=Next Generation Science Standards. Other abbreviations are consistent with those used in the NGSS: PS=Physical science; LS=Life science; ESS=Earth & Space science; ETS=Engineering, Technology, and Application of Science. Numbers following abbreviations indicate the disciplinary core ideas described in NRC, 2012).

Figure 1 shows the yearlong curriculum map for the sixth-grade instructional sequence, highlighting the progression of garden-based instructional units and activities as they were developed and aligned with the classroom curriculum. As sixth graders, the students in this study commenced SciLG programming in the spring term of 2015. The spring unit emphasized an extended investigation exploring how environmental and genetic factors impacted plant growth and survival. Through this investigation, students also grappled with connections between nature (genetic factors) and nurture (environmental factors). These learning experiences laid the foundation for deeper learning about epigenetics and genetics to be covered in high school science. By the end of the school year, students were able to analyze their own data in comparison to historical climate patterns. Each of the units gave students opportunities to engage in the practices of science and engineering, for instance, developing explanations and models using evidence.

### Culturally responsive pedagogy

Culturally responsive pedagogy begins with the assumption that if we are to be successful in teaching science to every child, we must reject the deficit approaches that some educators have historically brought to students in minority groups and instead start with an appreciation of culture (Settlage et al. 2017). This perspective views the multicultural, lived experiences of students as strengths, capitalizing on “the rich and varied cultural wealth, knowledge, and skills that diverse students bring to schools” (Howard 2012, p. 550). The essential elements of culturally responsive pedagogy include valuing students’ assets, connecting learning to students’ lives outside of school, fostering positive teacher-student relationships, and shifting the power dynamics between educator and learners.

For decades, STEM education has been a realm held exclusively for accelerated, advanced students, yet, when STEM is taught through

real-life explorations that require students to gather and analyze data; to create models; to make observations; to build, test, redesign, and redefine their ideas, all in order to discover a scientific concept or hidden truth…it is riddle-solving at its finest! (Hawkins 2014, p. 77)

Through active engagement, students solve problems and mysteries of the natural world, rather than simply memorize facts. Grappling with real-world issues challenges students to learn science by *doing* science (Hawkins 2014). By engaging students in scientific practices, teachers can help them connect science to real-world issues in their daily lives. Encouraging students to address authentic problems in their schools and communities allows them to explore their own ideas and questions as they apply their understandings of science to develop solutions (Chapman and Feldman 2017; Yager and Brunkhorst 2014).

This collective body of research points to the important role that educators play in stimulating students’ interests in science. In order to legitimize students’ diverse cultural understandings, a wide variety of pedagogical and inclusive strategies are used to help bridge academics with students’ everyday experiences. Teacher-student relationships are also critical (Brown and Crippen 2017; Cutter-Mackenzie 2009; Ladson-Billings 1995). Through close and caring relationships, teachers validate and build on students’ prior knowledge and experience, making science relevant and meaningful. When students are supported in these endeavors by caring educators, they become more engaged and motivated to learn. These types of activities and relationships can help students feel more competent and welcome in the communities and practices of science, and make connections between science and their own interests and daily lives, which in turn increase their academic engagement (Chittum et al. 2017; Connell and Wellborn 1991; Deci and Ryan 1985; Fredricks et al. 2004; Ryan and Deci 2016, 2017; Skinner et al. 2008, 2009; Skinner et al. 2012).

### Self-determination theory

We used a motivational model which combines culturally responsive pedagogy with principles from self-determination theory (SDT, Deci and Ryan 1985; Ryan and Deci 2017; Skinner et al. 2008; Skinner and Pitzer 2012) to provide a useful, research-based framework for analyzing program impacts of SciLG on motivation and engagement and identify the specific factors that support students in minority groups in STEM (Skinner et al. 2012). From the macro-theory of SDT, this model draws on the basic needs mini-theory (Reeve, 2012) as instantiated in the self-system model of motivational development (Connell and Wellborn 1991; Skinner, Wellborn and Connell, 1990; Skinner and Pitzer 2012) and applied to learning in garden-based education (Skinner et al. 2012). When examined through the lens of SDT, culturally responsive garden-based education shows potential for increasing the quality of students’ academic engagement by supporting their basic needs for autonomy, relatedness, and competence (Deci and Ryan 1985; Reeve, 2012; Skinner et al., 2009, 2012).

**Fig. 2:**
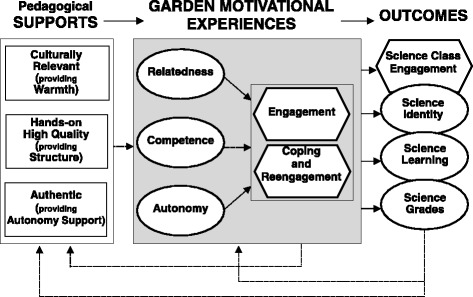
A motivational model of science learning in the garden, in which culturally relevant and supportive pedagogical contexts foster high-quality motivational experiences in the garden (i.e., where students’ needs for relatedness, competence, and autonomy are met and they are fully engaged in garden activities), which in turn promote students’ engagement in science class as well as their science identity, learning, and grades. Solid lines indicate theoretical connections examined in the current study. (Adapted from Skinner et al. 2012)

As depicted in Fig. 2, this model highlights both curricular and interpersonal factors that help students develop a positive academic identity for science and to engage, persist, and succeed in science. First, they must construct a set of self-appraisals or convictions about themselves, namely, that they are competent or efficacious; that they are related to or belong in communities of science, like school gardens; and that they are autonomous and take ownership for their own academic progress. These self-perceptions may be especially important for students in minority groups in academic and science settings where such students have often been subject to the majority culture’s doubts about whether they are sufficiently “talented” for academic and science careers. Such societal assumptions can perpetuate stereotype threats (Elliott 2015) and lead students to feel incompetent or unwelcome in science, which can prevent them from developing feelings of ownership, commitment, and identification in these fields (Walton and Cohen 2007). In contrast, *positive* self-appraisals, along with authentic and interesting academic tasks, support students’ engagement with learning activities and their resilience in the face of challenges and setbacks (Skinner et al. 2009, 2012). These motivational resources, in turn, contribute to success in science as well as in other academic domains (Fredricks et al. 2004; Wentzel 1997).

### Gardens as milieu for learning

Garden-based educational programs show promise as meaningful, culturally responsive, real-life, supportive contexts for promoting students’ engagement and other important academic outcomes (Blair 2009; Elliott 2015; Fusco 2001; Gaylie 2011; Moore 1997; Ozer 2006; Williams and Dixon, 2013). A recent meta-analysis and synthesis of 48 research studies on garden-based learning from 1990 to 2010 showed positive effects on a variety of academic outcomes including science, language arts, and mathematics and on a variety of outcomes that indirectly support academics including development of self-concept, change in eating habits, and positive environmental attitudes (Williams and Dixon 2013). The majority of gardens examined in these studies were integrated with science classes (Klemmer et al. 2005a, 2005b; Rahm 2002; Smith and Motsenbocker 2005). Of the 40 studies assessing direct learning outcomes, 33 (83%) found positive effects. Fifteen studies using garden-based learning measured science outcomes, of which 14 showed positive effects. For example, in one study in Temple, Texas, that used a sample of 647 students in grades 3–5 in seven elementary schools, Klemmer et al. (2005b) found that for those students who participated in a hands-on school gardening program, science achievement scores were higher than for those students who did not participate. They concluded that constructivist, hands-on learning is a main feature of school gardens; hence, they “serve as living laboratories in which students can see what they are learning and in turn, apply that knowledge to real world situations” (p. 452). As explained by Williams and Dixon (2013), “Soil chemistry, plant taxonomy, plant parts, flower dissection, water properties, seed germination and variety of seeds, insects and other wildlife, ecology and environmental horticulture, and insects and diseases” were among the themes represented in the research studies that they analyzed (p. 219).

Taken together, findings showed the potential of garden programs for benefitting academic and academic-related outcomes, especially in science. The integration of garden-based activities may likely be not only an important ingredient for science learning, but may also shape students’ engagement and enthusiasm for science in the regular classroom. Cumulatively, engagement in the gardens and in science class may serve as a mechanism of personal transformation in a student’s academic identity, convincing students in minority groups that they are “the kind of person” who is needed and who can succeed in science (Saxton et al. 2014; Skinner et al. 2012).

Garden-based programs grounded in activities and teaching practices that are culturally, motivationally, and developmentally responsive have the potential to bolster engagement in science and other core subjects and may help counteract motivational declines typically observed during the transition to middle school (Eccles et al. 1993; Gottfried et al. 2001; Wigfield et al. 1991; Wigfield et al. 2015). Helping to mitigate or reverse motivational declines is especially valuable for students who might otherwise be at risk for underachievement and drop-out. Bringing together tenets of SDT and culturally responsive pedagogy, garden-based education can provide authentic learning activities, promote positive teacher-student relationships, and nurture students’ sense of belonging and connection to place, narrowing gaps in opportunities for relevant, high-quality learning for historically underserved students (Elliott 2015).

### Purpose of study

This study examines student motivation, achievement, and science identity based on the experiences of racially and ethnically diverse students, at two low-income urban middle schools, who participated in *Science in the Learning Gardens* (SciLG) program. The primary goal is to determine whether students’ motivational experiences in SciLG activities are linked to important science outcomes, both concurrently and across school years. Building on earlier work, we were guided by a motivational model of science learning in the garden (see Fig. 2; Skinner et al. 2012), in which culturally relevant and supportive pedagogical contexts foster high-quality motivational experiences in the garden, that is, where students’ needs for relatedness, competence, and autonomy are met and they are fully engaged in garden activities. These motivational experiences, in turn, promote students’ engagement in science class as well as their science identity, learning, and grades. These science outcomes in turn feed back into subsequent contextual supports and motivational experiences in the garden.

### Motivational experiences in the gardens

To capture students’ motivational experiences while participating in SciLG activities, this study relies on a set of theoretically guided survey measures based on SDT (Connell and Wellborn 1991; Saxton et al. 2014) that tap students’ self-system perceptions, engagement, and coping/persistence in garden activities (Skinner et al. 2012, in press). Together, these are the wholistic experiences and actions that SciLG is designed to facilitate. Because SciLG activities are hands-on, culturally relevant, and authentic, they should help students feel more competent and related in the gardens and more autonomous in their reasons for participating in garden activities. However, culturally informed and caring pedagogical techniques are only impactful to the extent that students feel as if they and students like them are welcome and valued while in the garden. Hence, measures of the three self-system perceptions (hereafter, SSPs) of competence, relatedness, and autonomy give information about whether the intended pedagogical aspects of the SciLG activities are actually received by students.

In a similar vein, the experiential, NGSS-aligned activities will only support students’ learning and motivation to the extent that students actually invest emotionally and behaviorally while participating in those activities. Thus, we also measured students’ reports of their own emotional and behavioral engagement and disaffection in the gardens, examining the extent to which students felt they were energized and enjoying themselves during activities and the extent to which they dedicated their full thoughts and efforts to SciLG tasks. Finally, to see whether the garden activities provided a venue for students to build their persistence and capacity to bounce back when encountering setbacks, students’ science teachers reported on each student’s capacity to re-engage in the face of day-to-day academic challenges.

Hence, we represented SciLG motivational processes as a whole by creating an aggregated variable which equally weighted SSPs, engagement (versus disaffection), and re-engagement in the garden. Conceptually, this aggregate corresponds exactly to the set of experiences posited to be the “active ingredients” in SciLG (see Fig. 2). Empirical support for combining these measures comes from a recent study (Skinner et al. 2012), which found that students’ feelings of competence, autonomy, and intrinsic motivation for gardening were associated with both student- and teacher-reports of student engagement in gardening activities, suggesting that, together, self-perceptions and engagement may comprise a package of motivational experiences in the garden.

#### Science engagement, learning, achievement, and identity

To capture important science outcomes, we selected four markers. To establish whether the quality of students’ participation in SciLG was linked to motivation for science in the more typical classroom setting, we examined links between SciLG and students’ effortful, energized participation with learning activities in science class, as captured by students’ reports of their emotional and behavioral engagement and disaffection in science class. To see if SciLG activities were associated with students’ feelings of successful learning in science class, we used students’ reports of what and how much they learned in science. To check students’ perceptions of learning against their actual achievement, we targeted students’ term grades in science class. Finally, to see if SciLG activities seemed related to diverse students’ perceptions of themselves as people with interest and capacity to pursue science in the future, we used students’ reports of their science identity: being someone who belongs in science and who may want to pursue science in college or career.

### Research questions

Two research questions probed the linkages of SciLG motivational processes with science outcomes. The first examined whether the combined measure of overall motivational experiences in SciLG in the spring term was linked to sixth-grade students’ concurrent science engagement, self-reported learning, grades, and science identity, also collected in the spring. We hypothesized that SciLG motivational processes would significantly and positively predict each of the four science outcomes in the spring. The second research question examined whether motivational processes in the spring of sixth grade could predict the four science outcomes in the following fall term, when students were in seventh grade. We hypothesized that SciLG motivational processes in the spring of sixth grade would also significantly and positively predict all four outcomes in the fall of seventh grade.

## Methods

### Overview of Science in the Learning Gardens

Data were drawn from two highly diverse, Title I (low-income) schools with 82% of students qualifying for free and reduced lunch, where all sixth-grade students took part in SciLG garden-based education classes in the spring term of 2015 and again, as seventh graders, in the following fall 2015 term. Students’ three science teachers were supported by graduate assistants from Portland State University, integrating science themes in the garden curriculum with hands-on activities in the school gardens. Every week, six classes of 24–30 sixth-grade students per class came to the gardens with their classroom science teacher, for a 50–90-min block. Portland State University students managed the day-to-day maintenance of the gardens; with the teachers, they integrated the science curriculum aligned with NGSS (see Additional file 1) for use in the gardens, as the middle school students rotated through various stations for a variety of topics engaging with garden learning in small groups. The garden served as an extension of the schools’ classroom. Besides acquiring basic gardening skills, students discovered their connections to the place-based flora and fauna, studied science with special focus on the NGSS curriculum for the day, learned to compost, created art, and shared cultural stories about food and gardening. Team building was fostered through collaborative garden projects.

### Participants

All 209 sixth graders at the two schools were invited to participate in the study. Parental consent was received for 129 of the students (61% return rate). Of these students, 113 students had data on at least one predictor and one outcome variable and were included in the study. Students were 59% female and were ethnically and racially diverse (25% Asian, 2% Black, 26% Latino/Hispanic, 27% White, 18% Multiple ethnicities, 1% other ethnicities). Students were also linguistically and culturally diverse: English was not the primary home language for 51% of students, which was indicative of the high number of immigrant families at these schools. The most common home languages spoken were Spanish, Vietnamese, Russian, and Chinese; parental consent materials were translated into these languages.

### Design, procedures, and measures

Data for this study were collected in 2015, during the spring of students’ sixth-grade year (in May) and in the fall of their seventh-grade year (in November). Student surveys were administered in science classes by trained researchers and university student research assistants, using laptop computers and tablets. Science teachers completed paper-and-pencil surveys. Students and teachers rated their agreement with Likert-type survey items on a rating scale from 1 to 5 (where 1 was “not at all true for me/this student” and 5 was “very true for me/this student”). Negative items were reverse-coded.

#### Motivational processes in the garden

The predictor variable was a combined measure of students’ overall experiences in SciLG gardening activities. Scales assessing students’ reports of their *garden self-system perceptions* and *garden engagement* and teachers’ reports of students’ *garden re-engagement* (see Fig. 2) were adapted and expanded from a suite of garden motivation measures (Skinner et al. 2012, in press), employing the same procedures that were used to create the original measures. All items appear in Additional file 2.

*Garden self-system perceptions* (SSPs) were computed by averaging students’ scores from scales measuring students’ competence, relatedness, and autonomy in relation to garden activities. *Competence* was measured using seven items tapping students’ feelings of having the capacity to succeed in the garden-based activities (e.g., “I can do good work in the garden”; “I just can’t seem to do the right thing in the garden,” reverse-coded). *Relatedness* was measured using six items tapping students’ feelings of belonging (Osterman 2000) and acceptance in the garden (e.g., “I feel like a real part of the garden”; “Sometimes I feel like I don’t belong in the garden,” reverse-coded). *Autonomy* was measured using four items that captured students’ sense of doing their garden activities for personally motivated (rather than externally motivated) reasons (e.g., “Why do I garden? It makes me feel like I am doing something good for the environment,” “Because in the garden, I have noticed that I am learning important things”).

*Garden engagement* was measured using a 12-item scale capturing students’ perceptions of their energized and effortful participation in the gardens, assessing both emotional and behavioral participation (e.g., “I look forward to the time we spend in the garden,” “I try hard to do well in the garden”) versus their disaffection (e.g., “Gardening is not all that fun for me,” or “When we are in the garden, I can’t wait for it to be over,” reverse-coded) when participating in SciLG activities. *Garden re-engagement* was measured with two teacher-report items. Teachers rated their observations of each student as either persisting or giving up when encountering everyday challenges in gardening activities (e.g., “When faced with a difficult garden assignment, this student just keeps at it”).

##### Inter-correlations among garden SSPs and engagement

As expected, measures of students’ reports of their SSPs and engagement in the garden were positively and significantly inter-correlated (ranging from .58 to .75), as were inter-correlations with teacher-reports of student re-engagement in the garden (ranging from .33 to .42), which allowed these measures to be combined into an aggregate indicator of students’ overall motivational experiences in the garden.

#### Science outcomes

To explore how students’ experiences in SciLG impacted their participation in science, we included measures of four specific science outcomes.

#### Learning in science class

A seven-item scale was adapted from a measure of science learning (Skinner et al. 2012). Students reported on what they learned about science (e.g., “We learned how to experiment, observe, and measure,” “I learn how science can help solve real problems”) and how much they felt they learned in science class (e.g., “We learn lots of cool stuff in science class,” or “I do not learn much in science,” reverse-coded).

##### Engagement in science class

Students’ energized, effortful participation in science class was measured using a 12-item scale adapted from Skinner et al. (2012). Items assessed both emotional and behavioral engagement (e.g., “I pay attention to my science teacher,” “Working on science is interesting”) and disaffection (e.g., “When we work on something in science class, I feel bored,” “I don’t try very hard in science”).

##### Science identity

A nine-item scale was adapted from a measure of science academic identity (Saxton et al. 2014). Students reported their sense of being somebody who would be capable and accepted in the field of science (e.g., “I am the kind of person who belongs in science,” or “People like me do not get jobs in science,” reverse-coded) and their interest in pursuing a career or studies in science (e.g., “I would like to have a job that uses science”).

#### Science grades

Students’ spring and fall grades in science class were obtained from school records for the last semester in spring in the sixth grade and the first semester of fall in seventh grade. These were re-coded to a standard 0–4 scale where A = 4 and F = 0.

## Results

### Descriptive statistics

Means, standard deviations, and scale reliabilities for study constructs can be found in Table 1. Measures demonstrated satisfactory reliability: Cronbach’s alphas for all scales were at least .90. Overall mean levels suggested a generally positive experience for students, both in the garden and in science classrooms. The combined measure of students’ motivational experiences in the garden, as well as each of its subcomponents, in spring of sixth grade averaged about a 4 on the 1–5 scale, indicating that students and teachers both reported that positive items were “mostly true” (4) and negative items were only “a little true” (2). In terms of science outcomes, mean levels on all four variables also indicated generally positive processes during both spring of sixth grade and fall of seventh grade. Students endorsed as “mostly true” statements about their energized, effortful engagement in science class, about learning a lot in science, and about their science identity as someone who would belong in, and be interested in pursuing, a career or studies in science. Students earned an average “B” grade in science in both spring of sixth grade and fall of seventh grade.

**Table 1:** Descriptive statistics and internal consistencies for spring of sixth grade and fall of seventh grade

Construct	No. of items	Cronbach’s *α*	Mean	Standard deviation
Predictor variable (spring of 6th grade)				
Overall motivational experiences in the garden	31	.94	3.80	.76
Competence, autonomy, and relatedness	–	–	3.56	.84
Garden engagement	–	–	3.90	.80
Garden re-engagement (T-R)	–	–	3.94	1.16
Outcome variables (spring of 6th grade)				
Engagement in science class	12	.92	3.81	.92
Science learning	7	.92	3.83	1.03
Science grades	–	*–*	3.07	.94
Science identity	9	.92	3.20	1.03
Outcome variables (fall of 7th grade)				
Engagement in science class	12	.91	3.82	.82
Science learning	7	.90	3.80	1.00
Science grades	–	*–*	3.37	.99
Science identity	9	.90	3.40	.89

Total *n* = 113. Science grades ranged from 0 (“F”, lowest) to 4 (“A”, highest). All other constructs could range from 1 (“not at all true”) to 5 (“very true”). Negative items reverse-coded. Reliabilities calculated using SPSS v. 23; all other analyses conducted in MPlus 6.0, using Full-information Maximum Likelihood method to estimate missing data

*T-R* teacher report

### Inter-correlations among science outcomes in spring of sixth grade and fall of seventh grade

Correlations among study constructs are presented in Table 2. As expected, the four science outcomes were positively and significantly inter-correlated, indicating that they were, for the most part, inter-related and yet distinguishable, capturing complementary facets of students’ science experiences. Students’ self-reported science outcomes showed moderate inter-correlations, suggesting a relatively cohesive experience in both spring of sixth grade and fall of seventh grade, in which students who were engaged in their science class also felt a positive science identity and sense of learning science. Correlations between student-report science outcomes and science grades were, as expected, weaker, and in fall of seventh grade, they did not reach significance. However, intra-construct stabilities from spring of sixth grade to fall of seventh grade were moderate, indicating that students’ science experiences were similar, but not identical, at those two time points, so that the two research questions (examining the prediction of outcomes in the spring of sixth grade and in the fall of seventh grade) did seem to investigate distinct aspects of students’ experiences.

**Table 2:** Inter-correlations within time points and cross-time stabilities for study constructs

Constructs	Overall motivational experiences in the garden, spring of 6th grade	Engagement in science class	Science learning	Science grades	Science identity
Predictor variable (spring of 6th grade)					
Overall motivational experiences in the garden	–	.51	.53	.22^*^	.48
Outcome variables					
Engagement in science class	.71	*.44*	.82	.15^*ns*^	.60
Science learning	.72	.84	*.47*	.07^*ns*^	.58
Science grades	.31^**^	.24^*^	.24^*^	*.55*	− .02^*ns*^
Science identity	.59	.57	.64	.31**	*.36*

Total *n* = 113. All analyses conducted in MPlus 6.0, using Full-information Maximum Likelihood method to estimate missing data. Correlations for outcome variables in spring of 6th grade are below the diagonal. Correlations for outcome variables in fall of 7th grade are above the diagonal. Cross-time stabilities (fall-spring correlations) for each dependent variable are italicized on the diagonal. All coefficients are significant at *p* < .001 unless otherwise indicated

*p < .05

**p < .01

*ns* not significant

### Garden experiences in spring of sixth grade and science outcomes in spring of sixth grade and fall of seventh grade

As can also be seen in Table 2, the combined measure of motivational processes in the garden in spring of sixth grade was significantly and positively correlated with all science outcomes. Correlations among constructs measured at the same time point (cross-sectional analyses) were generally stronger than spring-to-fall correlations (longitudinal analyses), and correlations among constructs reported on the survey were stronger than the correlations between survey-report constructs and grades. Motivational processes in the garden in spring of sixth grade showed moderate concurrent correlations with student-report outcomes and a weaker correlation with science grades. As expected, correlations of motivational processes in the garden in spring of sixth grade and science outcomes in the fall of seventh grade showed a slightly weaker but otherwise similar pattern, with moderate correlations of student-report outcomes and a weaker correlation with science grades.

### Motivational experiences as concurrent predictors in spring of sixth grade

To answer the first research question, a series of regression analyses investigated whether students’ motivational processes during SciLG gardening activities in the spring of sixth grade seemed to transfer back into the science classroom. As hypothesized, motivational processes in the garden in the spring were significantly and positively connected with each of the four spring-term science outcomes (see Table 3). Students with more positive motivational processes in the garden reported significantly higher levels of engagement and learning in science class, as well as a more positive identity in science. They also received higher grades in science at the end of the year.

**Table 3:** Overall motivational experiences in the garden as a predictor of concurrent and later science outcomes

Predictor (spring of 6th grade): overall motivational experiences in the garden	Pairwise *n*	*β*	*SE*	*t*	*R* ^2^
Outcome variables (spring of 6th grade)					
Engagement in science class	97	.65^***^	.06	10.05	.61
Science learning	88	.70^***^	.06	10.98	.55
Science grades	111	.29^**^	.10	2.75	.08
Science identity	103	.59^***^	.08	7.76	.34
Outcome variables (fall of 7th grade)					
Engagement in science class	82	.57^***^	.10	5.60	.26
Science learning	68	.56^***^	.11	5.01	.26
Science grades	101	.23^*^	.11	2.12	.04
Science identity	90	.52^***^	.10	5.21	.21

Total *n* = 113. Overall motivational experiences in the garden is a combination of students’ appraisals of relatedness, competence, and autonomy in the garden; their self-reported engagement in the garden; and teacher-reports of students’ persistence and re-engagement in garden activities. Standardized regression coefficients are shown from regressions conducted in MPlus 6.0, using FIML to estimate missing data. All analyses controlled for spring science teacher

*p < .05

**p < .01

***p < .001

### Garden experiences in spring of sixth grade as predictors of science outcomes in fall of seventh grade

Another series of regression analyses were used to examine our second research question, testing whether positive effects associated with garden experiences in the spring persisted over the summer into the next fall. Again, as hypothesized, garden experiences in the spring of sixth grade positively and significantly predicted all four science outcomes in the fall of seventh grade (see Table 3). Students with more positive motivational processes in the garden in spring also reported higher levels of engagement in science class the following fall, as well as a more positive science identity. They also reported higher levels of science learning and received higher grades in science during their first term in seventh grade.

## Discussion

When examining the study’s first cohort of students in the spring of their sixth-grade year, descriptive statistics suggested that SciLG activities were successful in promoting high-quality motivational processes in the garden, with students and teachers generally endorsing positive items and disagreeing with negative items when asked about students’ self-perceptions, engagement, and re-engagement in gardening activities. Findings related to the first research question showed that a combined measure of these motivational processes in SciLG gardening activities was a significant and positive predictor of science-class engagement, science learning, grades, and science identity. That is, when students reported feeling more competent, related, autonomous, and engaged in the garden and their teachers reported that students re-engaged more after difficulties in the garden, those same students reported higher levels of energized and effortful participation with science class activities. Students with more positive motivational processes in the garden also showed higher levels of self-reported science learning as well as higher science grades, indicating both a better sense of learning about science and better actual performance in science class. Finally, when students had more positive motivational processes in the garden, they reported a stronger science identity, indicating more interest in pursuing science as a career or field of study and an increased identification as someone who could be accepted and successful in those pursuits. These effects offer support for the idea that students’ experiences with SciLG activities in the garden may transfer back into the science classroom (via grades, learning, and motivation) and help students identify with the community of science.

Findings for the second research question showed that students’ SciLG gardening experiences in spring of their sixth-grade year also significantly predicted all four science outcomes during the following fall when students started seventh grade. That is, despite adjourning for summer vacation and entering new science classrooms, it seemed that students’ spring gardening experiences may have served as positive resources for their science motivation, learning, achievement, and science as they began the next school year. In support of our second hypothesis, when students experienced higher levels of competence, relatedness, autonomy, engagement, and re-engagement in the garden in the spring of sixth grade, they tended to be more engaged with learning activities in their seventh-grade science classrooms, as well as reporting learning more science content, reporting a more positive identity as somebody who belongs in science as a field, and actually earning better grades in science class.

These findings from the first phase of a three-year longitudinal study suggest that learning gardens show promise in having the potential to positively impact students’ success in, and connection to, science. This research provides evidence that participation in a culturally responsive, NGSS-aligned garden-based program not only fostered students’ positive views of themselves in the garden and their engagement and persistence in the gardens, but also their engagement, learning, grades, and identity in their science classes. This empirical evidence supports the assumptions embedded in SciLG—specifically that involving middle school students in authentic, real-world endeavors that have cultural and personal relevance beyond school will not only be engaging, but will also help students learn science with understanding.

### Limitations and future research

Although promising, this study has several limitations. First, the study is not experimental nor does it control for prior levels of outcome variables; therefore, results are correlational rather than causal. It is plausible that students’ experiences in the garden are supporting their participation in science class, but we think it likely that effects run in both directions. Students who enjoy and are engaged in science class are also more likely to enjoy and engage in science in an alternative venue, namely the gardens. Future studies that compare students who participated in SciLG to similar students who did not would provide more definitive evidence of the positive effects of the program. By the same token, future studies that examine students’ experiences in the program as a predictor of *changes* in science outcomes over time would provide better estimates of how such experiences contribute to the *development* of science motivation, learning, and identity as students move through middle school. In ongoing studies, we are following this cohort into their seventh- and eighth-grade years, with a focus on detecting the processes by which SciLG gardening activities might support improvements in students’ motivational experiences and science outcomes. These data will also allow us to investigate the cumulative effects of students’ participation in SciLG—examining, for example, whether gardening experiences in sixth grade can predict science outcomes in seventh grade *over and above* the effects of the quality of the learning activities students are experiencing concurrently during their seventh-grade year in the garden.

A second set of limitations is based on sample selection and size. As with all studies, students were included in these analyses only if their parents and/or guardians gave them permission to provide data for the study. Hence, the generalizability of the findings to students without such permission is uncertain, and it is unclear whether these students would benefit as much from their experiences in the garden. The relatively small sample size may also have created potential problems. The study may have been underpowered to detect effects, which could explain the lack of a significant connection between student self-reported science outcomes and science grades in fall of seventh grade and the low *R*^2^ values in the regressions involving science grades at both time points. If so, then, this suggests that the current findings likely represent a conservative estimate of the connections between motivational processes in SciLG and science outcomes.

A third limitation of this study resides in the measures. We used a composite predictor variable to capture the overall set of motivational processes students experience in the garden as a whole, but this aggregate did not allow us to examine the structural relations among these processes or to distinguish whether specific aspects of garden experiences predict particular garden outcomes. For example, it may turn out that students’ sense of belonging is more important than their feelings of competence in the gardens or that enthusiastic engagement in SciLG is the key to improving student learning in science. Future studies can examine the individual or unique effects of these inter-related motivational processes separately to see if they are all essential ingredients or if some seem more important than others. Studies can also use structural models to test the proposed connections among these aspects of student experiences, as depicted in Fig. 2, examining, for example, whether students’ feelings of relatedness, competence, and autonomy in the gardens are each unique predictors of their engagement and re-engagement in garden-based activities. At the same time, however, it is important to note that the theoretical frameworks guiding this project, namely culturally responsive pedagogy and self-determination theory, suggest that programs will need to be concerned with supporting *multiple* student needs—for relatedness, competence, and autonomy—if they are going to counteract societal stereotypes and meet students' motivational and cultural needs.

### Theoretical and educational implications

Consistent with research on positive academic outcomes of school gardens (Williams and Dixon 2013) this study suggests that garden-integrated science activities have the potential to rekindle student engagement and motivation for learning. At the same time, findings from the current study build on this work in two important ways. First, this study targets an age/grade group for whom very little research and garden-based programming exist, namely, middle school students. The majority of studies and curricula for garden-based programs are concentrated on third to fifth grades (see Williams and Dixon 2013 for a review). Few middle school curricula have been developed or tested, perhaps because schools realize that the middle grades are a key window for students’ science learning and are concerned that garden-based programs may not be sufficiently rigorous to ensure middle schoolers’ success and readiness for high school science. The second contribution of this project directly addresses such concerns, by fully integrating SciLG curricula with NGSS, so that instructional leaders do not have to choose between the rigor of NGSS and the motivational enhancement of garden-based learning activities. Such integration allows teachers to approach deep science learning through learning activities that middle schoolers find meaningful and fun.

The study also makes contributions to the theoretical frameworks that underlie this project. On the one hand, SDT contributes to approaches focused on culturally relevant pedagogy and the view that certain motivational needs and experiences are basic to all humans and therefore, universal. These include the need to belong and feel welcome, to feel efficacious, and to be respected and autonomous in one’s own learning. Cultural views suggest to SDT that societal stereotypes and differential opportunity structures make it difficult for students from minority and low-income backgrounds to get those needs fulfilled in the typical classrooms to which they are consigned, and especially in science classrooms. This study is encouraging in that it shows that when students are given the opportunity to engage in high-quality science learning activities, they participate enthusiastically and do well in the gardens and in science class.

By bringing together learning gardens, SDT, and culturally relevant pedagogies, this project intends to deepen our understanding of how to work with students during years that are crucial to their future success and decisions about science careers, when their motivation for school (and for science and math in particular) are typically declining (Vedder-Weiss and Fortus 2012). It suggests that, with an overarching sensitivity to students’ cultural strengths and lives outside of school, garden-integrated curricula can be crafted to meet schools’ needs for rigorous science activities as well as students’ needs for supportive, interesting, authentic, and relevant opportunities to participate in the community of science. Future research as the cohort continues through middle school will examine whether participation in this program can reduce or reverse motivational declines while helping prepare students for success in high school science and beyond.

## Conclusions

As concern for social justice is increasing—based on the achievement gap among African-American, Black, Native-American, Hispanic, and Latino students and their White and Asian peers—the growing school garden movement provides an opportunity to tip the scales by engaging students in authentic, real-world learning of science and cultivating their interests in science with holistic garden-based learning (Williams and Brown 2012). This study highlighted the role of students’ views of themselves as competent, related, and autonomous in the garden, as well as their engagement and re-engagement in the garden, as potential pathways by which gardening activities can shape science motivation, learning, and academic identity in science. As articulated by Museus et al. (2011), there is a sense of urgency to ensure success in school and participation in science fields, particularly for students from racial and ethnic minority groups who have not been successful in science in traditional settings. This study provides preliminary support for the notion that learning in school gardens has the potential to promote science equity via the opportunity for students to experience different ways of learning science that are engaging and motivating, which in turn may promote students’ sense of science identity and science achievement. Participating in SciLG activities seemed to help diverse students not only engage more productively in science class, but also to think of themselves as individuals who could be successful and valued as contributors to the scientific community. Findings also lend support for the current motivational model, based on self-determination theory, as a means for capturing the “active ingredients” of SciLG activities. Together, the findings provide support for the SciLG program and school gardens more broadly as milieus for addressing equity via science identity and achievement.

## Additional files

Additional file 1: Garden Curricular Examples with Next Generation Science Standards. (DOCX 46 kb)

Additional file 2: Survey Measures. (DOCX 28 kb)
