# Supplementary material for: Science in the Learning Gardens (SciLG): a study of students’ motivation, achievement, and science identity in low-income middle schools
Source: Int J STEM Educ. 2018 Mar 26;5(1):8. doi: 10.1186/s40594-018-0104-9 (PMC6310407; doi:10.1186/s40594-018-0104-9)
Supplement: Supplementary file 1 — Garden Curricular Examples with Next Generation Science Standards. (DOCX 46 kb) [file 40594_2018_104_MOESM1_ESM.docx]

| **Additional file 1**  **Garden Curricular Examples with Next Generation Science Standards**  Next Generation Science Standards (NGSS) Middle School (MS) Performance Expectations for Life Science (LS), Earth and Space Science (ESS), and Engineering, Technology, & Applications of Science (ETS) addressed via Gardens, and examples of investigations (inquiry) and problem-solving (engineering design). | | | |  |
| --- | --- | --- | --- | --- |
| **Disciplinary Core Ideas (Framework)** | **Disciplinary Component Ideas (Framework)** | **Middle School (MS) Performance Expectations (NGSS)** | **Potential Examples in Gardens** |  |
| *Molecules to Organisms: Structures & Processes* | **LS1.B**  Growth & Development of Organisms | **MS-LS1-5.** Construct a scientific explanation based on evidence for how environmental and genetic factors influence the growth of organisms. | Investigate microclimates in the garden and the impact on growth of particular variety of plants; comparing fruit production of different strains/varieties of plants. |  |
|  | **LS1.C** Organization for Matter and Energy Flow in Organisms | **MS-LS1-6.** Construct a scientific explanation based on evidence for the role of photosynthesis in the cycling of matter and flow of energy into and out of organisms. | Explorations of food webs and matter flowing from air to plant to soil and back Develop a molecular model of the complementary processes of plant photosynthesis and respiration. |  |
|  |  | **MS-LS1-7.** Develop a model to describe how food is rearranged through chemical reactions forming new molecules that support growth and/or release energy as this matter moves through an organism. |  |  |
| *Ecosystems: Interactions, Energy, & Dynamics* | **LS2.A** Interdependent Relationships in Ecosystems | **MS-LS2-1.** Analyze and interpret data to provide evidence for the effects of resource availability on organisms and populations of organisms in an ecosystem. | Studies of plant growth rates/biomass production in comparison to planting density; water quantities; compost and nutrients; and other factors. |  |
|  |  | **MS-LS2-2**. Construct an explanation that predicts patterns of interactions among organisms across multiple ecosystems. | Observe and explain different relationships in the garden (e.g., ladybug and aphid; legumes and nitrogen-fixing bacteria). |  |
|  | **LS2.B** Cycle of Matter and Energy Transfer in Ecosystems | **MS-LS2-3**. Develop a model to describe the cycling of matter and flow of energy among living and nonliving parts of an ecosystem. | Construct visual models demonstrating carbon cycle, nitrogen cycle, and energy flow through the garden system. |  |
|  | **LS2.C** Ecosystem Dynamics, Functioning, & Resilience | **MS-LS2-5**. Evaluate competing design solutions for maintaining biodiversity and ecosystem services. | Cover-cropping and compost; water catchment and plant density; plant communities to attract beneficial insects. |  |
|  | **LS2.D**  Biodiversity  and Humans |  |  |  |
| *Earth’s Systems* | **ESS2.A**  Earth’s Materials & Systems | **MS-ESS2-1**. Develop a model to describe cycling of Earth’s materials and the flow of energy that drives this process. | Include abiotic factors in models of nutrient cycles. | |
| *Earth & Human Activity* | **ESS3.C**  Human Impacts on Earth Systems | **MS-ESS3-3.** Apply scientific principles to design a method to monitor and minimize a human impact on the environment. | Student groups identify aspects of food production impacting environment (e.g. water consumption, run-off, burning fossil fuel, etc.), articulate connections between population growth and consumption, then design strategies to minimize and/or mitigate negative impacts. | |
|  |  | **MS-ESS3-4.** Construct an argument supported by evidence for how increases in human population and per-capita consumption of natural resources impact Earth’s systems. |  |  |
| *Engineering Design* | **ETS1.A** Defining & Delimiting Engineering Problems | **MS-ETS1-1**. Define the criteria and constraints of a design problem with sufficient precision to ensure a successful solution, taking into account relevant scientific principles and potential impacts on people and the natural environment that may limit possible solutions. | Any number of student- identified problems and design-based solutions. | |
|  | **ETS1.B** Developing Possible Solutions  **ETS1.C** Optimizing the Design Solution | **MS-ETS1-2**. Evaluate competing design solutions using systematic process to determine how well they meet criteria and constraints of the problem. |  |  |
|  |  | **MS-ETS1-3.** Analyze data from tests to determine similarities and differences among several design solutions to identify the best characteristics of each that can be combined into a new solution to better meet the criteria for success. |  |  |
