# Supplementary material for: Science in the Learning Gardens (SciLG): a study of students’ motivation, achievement, and science identity in low-income middle schools
Source: Int J STEM Educ. 2018 Mar 26;5(1):8. doi: 10.1186/s40594-018-0104-9 (PMC6310407; doi:10.1186/s40594-018-0104-9)
Supplement: Supplementary file 2 — Survey Measures. (DOCX 28 kb) [file 40594_2018_104_MOESM2_ESM.docx]

**Additional file 2**

**Survey Measures**

| Answered using a 5-point Likert-type rating scale: | | |
| --- | --- | --- |
| 1 = “not at all true for me/this student” | | 4 = “fairly true for me/this student” |
| 2 = “a little true for me/this student” | | 5 = “very true for me/this student” |
| 3 = “somewhat true for me/this student” | |  |
| **Perceived Competence in the Garden** | | |
| 1. | I am good at gardening. | |
| 2. | I can figure out how to make things grow. | |
| 3. | I am a big help in the garden. | |
| 4. | I can do good work in the garden. | |
| 5. | I don't have the brains to garden. (-) | |
| 6. | Gardening is too hard for me. (-) | |
| 7. | I just can’t seem to do the right thing in the garden. (-) | |
| **Autonomy in the Garden** | | |
| Why do I garden? | | |
| 1. | It makes me feel like I am doing something good for my school. | |
| 2. | It makes me feel like I am doing something good for the environment. | |
| 3. | Because it makes me feel like I am doing something good for my body. | |
| 4. | Because in the garden, I have noticed that I am learning important things. | |
| **Relatedness in the Garden** | | |
| 1. | I feel like a real part of the garden. | |
| 2. | The garden is a good place for students like me. | |
| 3. | I feel at home in the garden. | |
| 4. | Sometimes I feel like I don’t belong in the garden. (-) | |
| 5. | In the garden, I feel like an outsider. (-) | |
| 6. | When I am in the garden, I feel like I’m nobody. (-) | |
| **Garden Engagement** | | |
| 1. | I try hard to do well in the garden. | |
| 2. | When our gardening teacher asks us a question, I try to answer it. | |
| 3. | I listen carefully to our gardening teacher. | |
| 4. | When I’m in the garden, I feel good. | |
| 5. | Gardening is fun. | |
| 6. | I look forward to the time we spend in the garden. | |
| 7. | I don’t try very hard in the garden. (-) | |
| 8. | When we are gardening, my mind wanders. (-) | |
| 9. | When we are in the garden, I can't wait for it to be over. (-) | |
| 10. | When I’m in the garden, I am wet, dirty, not really interested in what we are doing. (-) | |
| 11. | Gardening is not all that fun for me. (-) | |
| 12. | I'd rather be doing just about anything else but gardening. (-) | |
| **Garden Re-engagement (teacher-report)** | | |
| 1. | When faced with a difficult assignment in the garden, this student just keeps at it. | |
| 2. | When faced with a difficult assignment in the garden, this student gives up quickly. (-) | |
| **Engagement in Science Class** | | |
|  | I work as hard as I can when I am in science class. | |
|  | When my science teacher asks us a question, I try to answer it. | |
|  | I pay attention to my science teacher. | |
|  | Working on science is interesting. | |
|  | Science is fun. | |
|  | I enjoy learning new things in science. | |
|  | I don’t try very hard in science. (-) | |
|  | When I’m in science, I think about other things. (-) | |
|  | When I am in science class, I can't wait for it to be over. (-) | |
|  | When we work on something in science class, I feel bored. (-) | |
|  | Science is not all that fun for me. (-) | |
|  | I'd rather be doing just about anything else but science. (-) | |
| **Science Learning** | | |
|  | I learn how to do science-- experimenting, measuring, observing, finding out new facts. | |
|  | I learn how to treat the environment better. | |
|  | I learn that science is everywhere-- like in the garden. | |
|  | I learn how science can help solve real problems. | |
|  | My science teacher teaches me new things all the time. | |
|  | We learn lots of cool stuff in science class. | |
|  | I do not learn much in science. (-) | |
| **Science Identity** | | |
|  | I am the kind of person who can succeed in science. | |
|  | I am the kind of person who belongs in science. | |
|  | For the job I want to have when I grow up, I need to know some science. | |
|  | I would like to have a job that uses science | |
|  | People like me would be good at a job that has to do with science. | |
|  | I am thinking of studying science in college. | |
|  | Science is important for my future career. | |
|  | Science doesn't have anything to do with me. (-) | |
|  | People like me do not get jobs in science. (-) | |

*Note*. Adapted and expanded from Skinner, Chi, & the LEAG, 2012. Negative symbol “(-)” indicates reverse-coded item.
